# ER-Resident Transcription Factor Nrf1 Regulates Proteasome Expression and Beyond

**DOI:** 10.3390/ijms21103683

**Published:** 2020-05-23

**Authors:** Jun Hamazaki, Shigeo Murata

**Affiliations:** Laboratory of Protein Metabolism, Graduate School of Pharmaceutical Sciences, the University of Tokyo, Tokyo 1130033, Japan; smurata@mol.f.u-tokyo.ac.jp

**Keywords:** proteasome, Nrf1, DDI2, bounce back, proteostasis

## Abstract

Protein folding is a substantively error prone process, especially when it occurs in the endoplasmic reticulum (ER). The highly exquisite machinery in the ER controls secretory protein folding, recognizes aberrant folding states, and retrotranslocates permanently misfolded proteins from the ER back to the cytosol; these misfolded proteins are then degraded by the ubiquitin–proteasome system termed as the ER-associated degradation (ERAD). The 26S proteasome is a multisubunit protease complex that recognizes and degrades ubiquitinated proteins in an ATP-dependent manner. The complex structure of the 26S proteasome requires exquisite regulation at the transcription, translation, and molecular assembly levels. Nuclear factor erythroid-derived 2-related factor 1 (Nrf1; NFE2L1), an ER-resident transcription factor, has recently been shown to be responsible for the coordinated expression of all the proteasome subunit genes upon proteasome impairment in mammalian cells. In this review, we summarize the current knowledge regarding the transcriptional regulation of the proteasome, as well as recent findings concerning the regulation of Nrf1 transcription activity in ER homeostasis and metabolic processes.

## 1. Introduction

Proteins are essential to the vast majority of cellular and organismal functions. Cellular protein levels are defined by the balance among protein synthesis, folding, and degradation, and the failure to accurately coordinate these processes leads to disease [1]. Under normal conditions, dietary amino acids constitute only approximately 20% of the amino acid supply required to build the proteins synthesized daily by an adult human in order to maintain a constant body weight [2]. The remaining ≈80% of amino acids come from the recycling following protein degradation [3]. Cells devote a significant fraction of their resources to surveying and maintaining protein homeostasis (proteostasis) even under optimal conditions because the accumulation of misfolded proteins represents a threat to proper cell and organismal function.

The ubiquitin–proteasome system (UPS) is the main machinery responsible for protein degradation in all eukaryotes. Substrate proteins are covalently modified by a small protein called ubiquitin that is activated by the coordinated actions of E1, E2, and E3 enzymes [4]. The 26S proteasome is a large protease complex that selectively recognizes and degrades ubiquitinated proteins in an ATP-dependent manner [5]. The UPS degrades thousands of short-lived proteins and regulator proteins, as well as damaged and misfolded proteins, in order to regulate various cellular functions including cell cycle, DNA repair, apoptosis, immune response, signal transduction, cellular metabolism, and protein quality control [6,7,8]. To perform these functions, the maintenance of appropriate proteasome activity is essential for cellular homeostasis.

Approximately one-third of all cellular proteins enter the endoplasmic reticulum (ER) and are exported to their final location. Proteins retrotranslocated from the ER to the cytosol are mainly degraded by the UPS in a process known as ER-associated degradation (ERAD) [9,10]. The UPS is central to the unfolded protein response (UPR), which is activated when unfolded or misassembled proteins accumulate in the ER, upon which the UPR leads to apoptosis if ER stress is not mitigated [11,12]. As such, dysregulation of proteasome function is associated with proteostasis in the ER.

**Figure 1:**
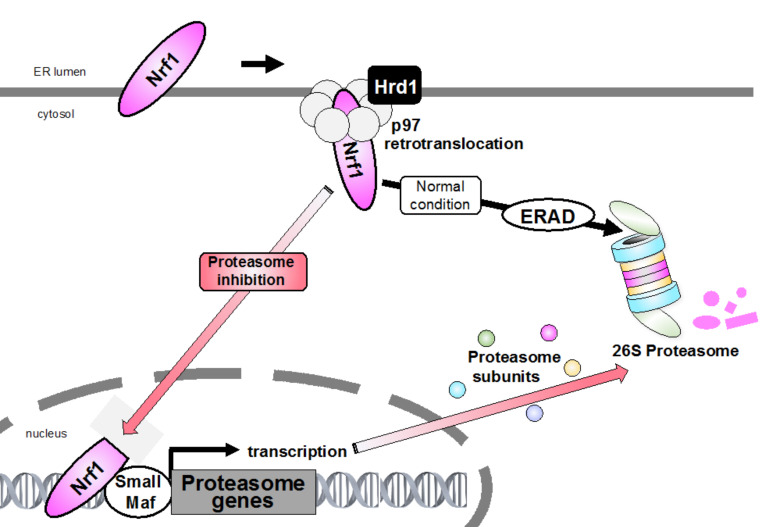
Bounce-back response. Endoplasmic reticulum (ER)-resident nuclear factor erythroid-derived 2-related factor 1 (Nrf1) is constantly degraded via ER-associated degradation (ERAD) machinery under normal conditions. During proteasome inhibition, accumulated Nrf1 translocates from the ER into the nucleus and promotes the expression of targets including proteasome subunit genes. This negative feedback mechanism of proteasome gene induction is called “bounce-back response”.

Proteasomal degradation is vital in all cells and organisms, and dysfunction or failure of which is associated with a diverse range of human diseases, including autoinflammatory syndromes and neurodegenerative diseases such as Alzheimer’s disease (AD), Parkinson’s disease (PD), and amyotrophic lateral sclerosis (ALS); on the other hand, an increase in proteasome activity is observed in cancer cells, emphasizing the importance of proper functional maintenance of the proteasome [13,14,15]. To ensure precise modulation of proteasome activity, the expression of proteasome subunits and proteasome assembly chaperones need to be adequately controlled. As the proteasome comprises 33 distinct subunits, proteasome biogenesis is elaborately regulated at several steps including transcription, protein assembly, and post-translational modifications. Nuclear factor erythroid-derived 2-related factor 1 (Nrf1), an ER-resident transcription factor, has recently been described as an essential key player in the maintenance of cellular redox and lipid and protein homeostasis; in particular, it plays a role in maintaining proteostasis by mediating the 26S proteasomal “bounce-back” response [16,17]. In this review, we focus on the transcriptional regulation of the proteasome, especially in terms of the recent findings regarding this topic (Figure 1).

## 2. ERAD and Proteasome

Proteins newly synthesized by ER-bound ribosomes are inserted into the ER [18,19,20]. Misfolded ER proteins are extracted from the ER into the cytosol by AAA^+^ ATPase p97/valosin-containing protein (VCP) for degradation by the proteasome. Hrd1, a major ER-resident E3, forms a complex with the ER-resident Sel1L (also known as mammalian Hrd3) and plays a central role in the degradation of a subset of ER-resident misfolded proteins [21]. It is known that the cystic fibrosis transmembrane conductance regulator (CFTR) is highly prone to misfolding, and most newly synthesized wild-type CFTR proteins are degraded by the ERAD [22,23]. In addition to soluble and membrane-bound ER proteins such as OS9, EDEM1, and inositol-requiring enzyme 1α (IRE1α), nuclear proteins such as the peroxisome proliferator-activated receptor-gamma coactivator 1β (PGC1β) have been identified as endogenous substrates for the Sel1L–Hrd1 complex [21]. Recent studies have shown that ERAD is not only activated as an ER stress response but also plays regulatory roles in many fundamental cellular processes by mediating the turnover of specific substrates [24,25,26,27].

In addition to misfolded proteins, ERAD degrades properly folded proteins in the ER. Human 3-hydroxyl-3-methylglutaryl-coenzyme A reductase (HMGR), an ER-resident integral membrane, limits the rate of mevalonic acid production and undergoes feedback-regulated degradation. When sterol levels are high, HMGR associates with insulin-induced gene (INSIG), which brings an ER-resident E3 gp78 close to HMGR, causing its ubiquitination and degradation by the proteasome [28]. Ligand-triggered degradation of the ER-bound IP3 receptor has also been observed in mammals [29]. Thus, ERAD-mediated finely tuned protein regulation is feasible and exists widely; however, the mechanisms underlying substrate recognition by ERAD remain poorly defined in mammalian cells.

The ERAD plays an essential role in maintaining protein homeostasis by alleviating the protein burden on the ER. Although a fraction of proteasomes are known to associate with the ER membrane [30,31], in situ cryo-electron tomography using the unicellular alga *Chlamydomonas reinhardtii* recently revealed that the cytosolic ERAD machinery is concentrated within 200 nm foci that contact specialized patches of the ER membrane [32]. These non-membrane-bound microcompartments consist of densely clustered 26S proteasomes surrounded by a loose cloud of cdc48 to enable efficient protein quality control.

The 26S proteasome is highly conserved in eukaryotes from *Saccharomyces cerevisiae* (budding yeast) to mammals, and is composed of a 20S core particle (CP) and a 19S regulatory particle (RP) that binds to either one or both ends of the CP [33,34]. In the CP, the α-ring and β-ring individually comprise seven distinct subunits, namely, the α1-α7 and β1-β7 subunits, respectively. These rings are stacked in an α-β-β-α topology to form a cylindrical shaped complex. The CP includes three proteolytically active subunits, namely, the β1, β2, and β5 subunits; the active sites of these subunits are located inside the cylindrical chamber. In response to pro-inflammatory cytokines such as TNF-α and interferon-γ, three catalytic subunits, β1i, β2i, and β5i, are induced to form the immunoproteasome. In cortical thymic epithelial cells, β5t are expressed abundantly and incorporated to the CP [35]. Recognition, unfolding, and translocation of substrate proteins towards the CP is performed by the RP [36,37]. The RP is divided into two subcomplexes, the base and lid. The base subcomplex contains six homologous AAA^+^ ATPase subunits (Rpt1-Rpt6) and four non-ATPase subunits (Rpn1, Rpn2, Rpn10, and Rpn13). The six AAA^+^ ATPases form a ring and participate in the unfolding and translocation of the substrates towards the interior cavity of CP in an ATP-dependent manner. Rpn10 and Rpn13 function as ubiquitin receptors [38,39]. Rpn1 also exhibits binding capacity towards ubiquitin; additionally, Rpn1 interacts with the ubiquitin-like domain, enabling the recruitment of shuttle factors that are composed of ubiquitin-like (UBL) and ubiquitin-associated (UBA) domains such as the human DNA repair protein hHR23. As the proteasome is a large and complex structure, proteasome assembly is regulated in a sophisticated manner [40,41]. CP and RP are separately constructed with the assistance of specific assembly chaperones. In CP assembly, PAC1–PAC4 mediate the α-ring formation, and afterwards, Ump1/POMP orchestrates β-ring formation on the α-ring and the dimerization of half-CPs to form mature CPs. In addition to the 19S RP, other proteasome regulators, such as proteasome activator (PA) 28αβ, PA28γ, PA200, ECM29, and PSMF1, are known to bind to the CP to play a significant role in diverse events through protein degradation.

## 3. Transcriptional Regulation of Proteasome Genes in Metazoan

**Figure 2:**
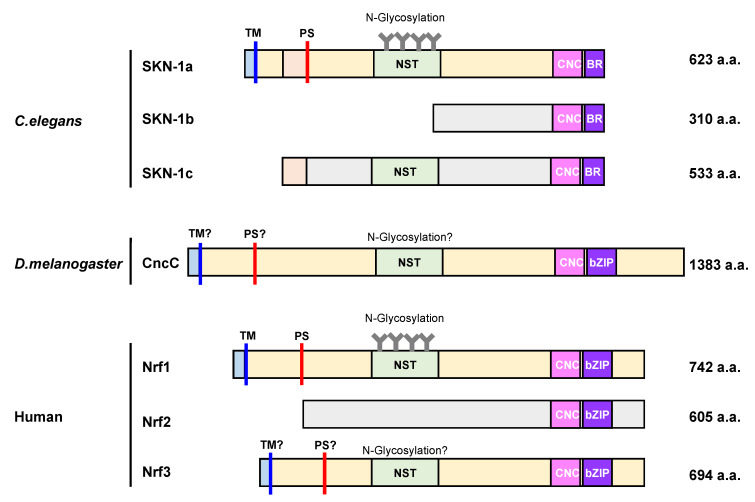
Domain organization of cap ‘n’ collar (CNC)-basic leucine zipper (bZIP) family proteins. The image depicts the domain organization of seven CNC-bZIP proteins, namely, SKN-1a, SKN-1b, SKN-1c, CncC, Nrf1, Nrf2, and Nrf3. CncC and Nrf proteins share a cap ‘n’ collar (CNC) and a basic leucine zipper (bZIP) domain in their C-terminus region, but SKN1 proteins only have a basic leucine domain (BR), which lacks the ZIP dimerization module. SKN-1a, CncC, Nrf1, and Nrf3 exhibit an N-terminal domain with a transmembrane (TM) region to anchor proteins to the ER membrane. Additionally, each of them, except for Nrf2, contain an Asn/Ser/Thr-rich (NST) domain, which is a target for N-glycosylation. The processing site (PS) is represented as red bars. To begin, the cytoplasmic region is colored in light blue, and the ER luminal region is colored in light yellow. *C. elegans: Caenorhabditis elegans*, *D. melanogaster: Drosophila melanogaster.*

Increasing the proteasome abundance when needed is important for sustaining protein degradation and cell viability, as the proteasome is essential for a broad range of housekeeping functions. As each proteasome subunit exhibits a distinct structure and specific function that cannot be fully substituted by the other subunits, the expression of all proteasome subunits is coordinately regulated at the transcriptional level [42]. In yeast, coordinated expression of proteasome genes is mediated by the transcription factor Rpn4, and an increased Rpn4 expression extends the lifespan of yeast [43]. A concerted increase in the expression of proteasome subunits in response to proteasome inhibition is also observed in *Caenorhabditis elegans* (*C. elegans*), *Drosophila melanogaster* (*D. melanogaster*), and mammals, which is similar to yeast even though Rpn4 is not conserved [44]. In these organisms, the Nrf/cap ‘n’ collar (Cnc) transcription factors, CncC (*D. melanogaster*), SKN-1 (*C. elegans*), and Nrf1 (mammal) are responsible for the expression of proteasome subunit genes, especially in the compensatory response (termed as the bounce-back response) [16,17,41,45] (Figure 2). Overexpression of certain proteasome subunits also upregulates 26S proteasome activity, resulting in lifespan extension in *C. elegans* and fruit flies, although its exact mechanism remains elusive [46,47,48,49,50,51].

### 3.1. Rpn4 (Yeast)

Rpn4 is essential for stress-induced proteasome expression in a negative feedback loop, as well as for constitutive proteasome expression in yeast [52]. Rpn4 binds to a specific sequence motif known as the proteasome-associated control element (PACE), which is present in the promoter regions of all proteasome subunit genes and in a few proteasome assembly chaperone genes [53]. Rpn4 is a protein with an extremely short half-life (t_1/2_ ≈ 2 min) as it is continually degraded by the proteasome [54]. Therefore, when proteasome function is compromised, Rpn4 accumulates and augments proteasome expression, leading to the recovery of proteasome function. The deletion of either Rpn4 or a PACE sequence in one of the proteasome subunit genes remarkably decreases proteasome activity and sensitizes cells to stress such as DNA damage and oxidation, demonstrating that the bounce-back response is essential for maintaining adequate proteasome activity [55]. In addition, Rpn4 abundance is regulated not only by its rapid proteasomal degradation but also transcriptionally by various stress-inducible transcription factors such as Yap1, Pdr1, Pdr3, and Hsf1, indicating that increasing the expression of proteasome subunits may be a common mechanism for adapting to diverse stress conditions [56].

### 3.2. SKN-1 (C. elegans)

Three protein isoforms (SKN-1A, SKN-1B, and SKN-1C) that show different expression patterns are generated by differential splicing and transcription start site utilization in *C. elegans* [57]. All three SKN-1 isoforms share an identical CnC DNA-binding domain at their C-termini, but they have different N-terminal sequences. SKN-1A, SKN-1B, and SKN-1C are expressed in all tissues, two sensory neurons, and the intestine, respectively. Genetic studies have revealed the importance of SKN-1, but the individual functions of SKN-1 isoforms are not fully understood [57]. Of the isoforms, only SKN-1A contains an N-terminal transmembrane domain, localizes to the ER, and is N-linked glycosylated, suggesting that SKN-1A is the counterpart of mammalian Nrf1. It has been shown that SKN-1A is activated by unfolded proteins even when the proteasome is not impaired [58]. SKN-1B is suggested to act in resistance to graphene oxide toxicity, although the molecular mechanism is elusive [59]. SKN-1C shows nuclear localization upon oxidative stress, suggesting that SKN-1C may have a function analogous to mammalian Nrf2 [60].

### 3.3. CncC (D. melanogaster)

The fruit fly transcription factor CncC regulates the expression of phase I and II enzymes, antioxidant proteins, and proteasome subunits [61]. Although CncC has a similar amino acid structure to SKN-1 and Nrf proteins, the distinct molecular mechanism for its activation is not verified. Moreover, CncC protein level is regulated by its direct interaction with Kelch-like ECH-associated protein 1 (Keap1). Thus, CncC is assumed to have a function analogous to both Nrf1 and Nrf2, and recent advances have shown that Cnc proteins have at least 16 variants [62].

### 3.4. Nrf Family Members (Mammals)

Vertebrates have four Nrf genes, namely, Nrf1, Nrf2, Nrf3, and nuclear factor erythroid 2 (NF-E2), implying that a diversification of the protein family occurred during vertebrate evolution. A recent study has revealed that Nrf1, Nrf2, and Nrf3 contribute to proteasome subunit gene expression with varying significance [63,64]. Nrf1 and Nrf2 are expressed across almost all tissues, whereas Nrf3 expression is predominantly placental and induced in several types of cancer cells such as colon adenocarcinoma [65]. Although Nrf2 was initially proposed to increase the expression of proteasome subunits, it was recently demonstrated that Nrf1 plays a pivotal role in the upregulation of proteasome gene expression and de novo proteasome synthesis in response to proteasome inhibition. Nrf1 has four isoforms, namely, Nrf1α, Nrf1β, and Nrf1γ, and Nrf1δ; however, it is unknown how each Nrf1 isoform contributes to regulate the expression of cytoprotective genes against various types of physiopathological stresses [66].

### 3.5. Other Transcription Factors that Regulate Proteasome Subunit Gene Expression

In mammals, several transcription factors such as nuclear transcription factor Y (NF-Y), forkhead box protein O4 (FOXO4), and signal transducer and activator of transcription 3 (STAT3) are known to regulate the expression of proteasome subunit genes [67,68,69]. NF-Y regulates the expression of genes, which contain one or more CCAAT boxes in their promoter region, such as six CP subunit genes (α2, α5, α7, β3, β4, and β6), five RP subunit genes (Rpt1, Rpt5, Rpt6, Rpn10, and Rpn11), and one assembly chaperone (p28). In pluripotent stem cells, FOXO4 regulates expression of Rpn6, which serves to a high proteasome activity. In several cancer cells, STAT3 promotes the expression of β-subunit genes and participates in epidermal growth factor (EGF)-induced proteasome upregulation. However, the mechanism of the expression of a particular set of subunits that successfully increases the quantity of completely assembled proteasomes remains unexplained.

## 4. Molecular Characteristics of SKN-1, CncC, and Nrf1 Proteins

**Figure 3:**
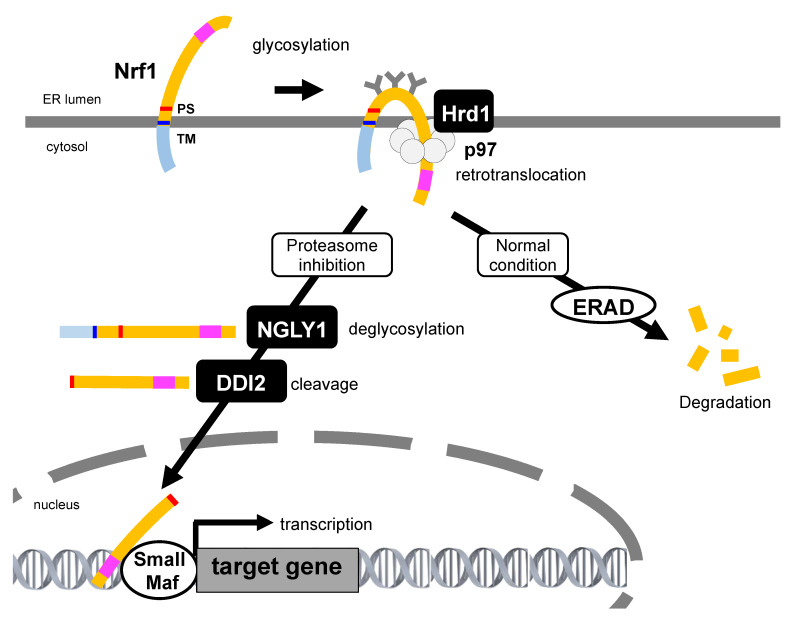
Nrf1 activation under the proteasome dysfunction. Under normal conditions, Nrf1 is ubiquitinated, retrotranslocated, and degraded by the ER-resident E3 Hrd1, p97, and proteasome, respectively. During proteasome inhibition, Nrf1 is glycosylated in the ER, ubiquitinated by Hrd1, and then retrotranslocated by p97. In the cytosol, polysaccharides attached to Nrf1 are removed by NGLY1 to generate the full-length Nrf1. Then, aspartic protease DNA damage-inducible 1 homolog 2 (DDI2) cleaves Nrf1 at the N-terminus region and produces the processed Nrf1. The processed Nrf1 translocates from the ER into the nucleus and promotes the expression of targets including proteasome subunit genes. Transcription activation domain is represented as a pink square. TM: transmembrane, PS: processing site.

SKN-1, CncC, and Nrf1 are among the CNC basic leucine zipper (CNC-bZIP) family of transcription factors that require the Maf dimerization partner to stably bind DNA [70,71]. The CNC domain is named after the *D. melanogaster* CnC gene and is a 43-amino acid region that flanks the N-terminal of the bZIP domain. In mammals, the CNC-bZIP family includes six members, namely, Nrf1, Nrf2, Nrf3, NF-E2 p45 subunit, BTB, and CNC homolog 1 (Bach1), and Bach2 [72]. The bZIP domain located near the C-terminus of Nrf1 is characterized by a 30-amino acid region enriched in arginine and lysine residues and a 40-amino acid helical region containing heptad repeats of leucine and hydrophobic residues. The former 30-amino acid region is responsible for DNA binding, whereas the latter 40-amino acid helical region is necessary for dimerization with Maf proteins such as MafF, MafG, and MafK. Additionally, as small Maf proteins bind to DNA through the bZIP domain, heterodimerization with a small Maf significantly promotes the DNA-binding efficiency of a CNC-bZIP protein [73]. Each SKN-1 isoforms binds to its cognate site on its own with an affinity comparable to that of a bZIP dimer, even though SKN-1 lacks the ZIP dimerization module [74,75,76]. A heterodimer of a CNC-bZIP protein and a small Maf protein binds to the antioxidant response element (ARE). The ARE comprises 5’-RTGACnnnGC-3’ (R = A or G) core sequence and is located in the enhancer and promoter regions of various genes that are mainly involved in phase 2 xenobiotic metabolism, drug transport, antioxidant response, and metabolic regulation. Recently, chromatin immunoprecipitation (ChIP)-seq data identified 5’-RTGACTCAGC-3’ as the consensus binding site of Nrf1 [77]. Notably, this binding site exists in the enhancer or promoter region of all 33 proteasome subunit genes, assembly factors, and alternative regulators. Therefore, Nrf1 coordinately activates the proteasome gene expression (Figure 3).

### 4.1. Control of Nrf1/SKN-1 Abundance

It is generally known that Nrf1 is co-translationally inserted into the ER membrane and that only a small portion of its N terminus is protruded into the cytosol, whereas the bulk of its polypeptide, including the transcriptional activation and DNA-binding domains, is embedded in the ER lumen. ER-resident proteins are generally degraded by the ERAD when they are misfolded. Interestingly, Nrf1 is constantly subjected to ERAD under normal conditions (Figure 2). In fact, ubiquitination by the ER-resident E3 Hrd1 and the ATPase activity of p97 are necessary for Nrf1 degradation.

Moreover, Nrf1 stability is regulated in the nucleus. Nuclear Nrf1 interacts with beta-transducin repeat-containing E3 (β-TrCP). β-TrCP forms a Skp1–Cul1–F-box (SCF) E3 complex that recognizes the DSGLS sequence in Nrf1, and ubiquitinates Nrf1 for degradation [78]. Recently, it was reported that Nrf1 stability is also regulated by O-linked N-acetylglucosamine (O-GlcNAc) modification in serine or threonine residues by the O-GlcNAc transferase (OGT), which is an enzyme that is highly active in many types of cancer. O-GlcNAcylated Nrf1 is unable to bind to β-TrCP. Additionally, nuclear Nrf1 is ubiquitinated by another SCF family E3 FBXW7, and is degraded by the proteasome [79]. Therefore, Nrf1 shows a short half-life (i.e., < 30 min), and its quantity is maintained at a low level under normal conditions. In contrast, USP15 physically interacts with Nrf1 and also stabilizes Nrf1 by removing its ubiquitin moieties, such that this transcription factor is activated to promote the expression of Nrf1-target proteasomal genes [80]. In *C. elegans*, the E3 substrate adaptor WDR-23 binds simultaneously to DDB-1, the cullin4 (CUL4) ortholog, and SKN-1, and is thought to trigger ubiquitylation and degradation of SKN-1 [81]. Thus, Nrf1 proteins might also be regulated by a highly conserved WDR-23 mammalian ortholog.

### 4.2. Retrotranslocation from the ER and Proteolytic Processing Are Required for Nrf1 Activation

The nascent Nrf1 is tethered to the ER membrane at its N-terminal transmembrane domain, and a large proportion of its polypeptide is N-glycosylated in the ER lumen [82]. The luminal domain of Nrf1 is rapidly transported to the cytosol by p97/VCP and is subsequently deglycosylated and proteolytically cleaved to produce the active form that translocates to the nucleus through proteasome inhibition (Figure 3, and Section 5 below). Therefore, abrogation of p97/VCP causes the accumulation of glycosylated Nrf1 and completely blocks the bounce-back response after proteasome inhibition [45]. A small fraction of Nrf1 deglycoproteins are likely sorted out of the ER membrane and transported to reside in the inner nuclear membrane so that they can gain direct access to their target genes [83]. Furthermore, when Ngly1 activity is insufficient, Nrf1 remains glycosylated and also associates with the ER membrane, such that Nrf1 accumulates outside the nucleus but is not able to properly regulate its target response genes [84]. Nrf1 escapes proteasome degradation and is accumulated as two main forms, both of which do not correspond to glycosylated Nrf1 under proteasome inhibition. The high molecular weight form (≈120 kDa) is anchored to the ER membrane, whereas the low molecular weight form (≈110 kDa), starting with the Leu104 residue which lacks the N-terminal transmembrane domain, localizes in the nucleus, and exhibits transcriptional activity. A recent study shows that Nrf1 is cleaved by the DNA damage-inducible 1 homolog 2 (DDI2) protease, resulting in the release of a proteolytic fragment of Nrf1 from the ER into the nucleus, activating the transcription of proteasome subunit genes. Recently, it was shown that DDI2-deficient or protease-dead DDI2 mutant MDA-MB231 human breast adenocarcinoma cells are more sensitive to carfilzomib-induced apoptosis [85], suggesting that DDI2 is significant to the bounce-back response.

**Figure 4:**
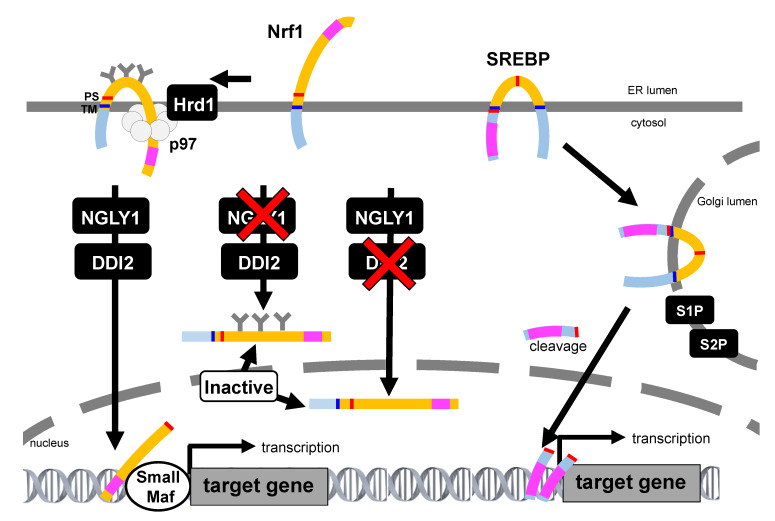
Comparison of Nrf1 and sterol regulatory element-binding protein (SREBP) activation pathway. The SREBP precursor is inserted into the ER, and both the amino-terminal transcription factor domain and the carboxyl-terminal regulatory domain are located in the cytoplasmic compartment. When the cellular demand for sterols rises, the SREBP precursor protein travels to the Golgi apparatus and is processed by site-1 protease (S1P) and site-2 protease (S2P). Then, the transcription factor domain released from the membrane enters the nucleus and induces the transcription of target genes. In contrast, the transcription activation domain of Nrf1 is initially in the ER lumen, which is topologically distinct to SREBP and other ER-resident transcription. Inhibition of NGLY1 results in the accumulation of glycosylated, unprocessed Nrf1. Inhibition of DDI2 leads the generation of a deglycosylated but unprocessed DDI2 that enters the nucleus while inactive. Thus, Nrf1 needs the proper sequential treatment for its activation. The transcription activation domain is represented as pink square. TM: transmembrane, PS: processing site.

It has also been shown that Nrf1 is proteolytically processed into a soluble, active C-terminal fragment that enters the nucleus and activates transcription upon partial proteasome inhibition. If proteasome activity is inhibited to a fuller extent, Nrf1 becomes incorporated into protein aggregates and becomes insoluble. These results imply that some proteasome functionality is necessary for Nrf1 processing; however, the exact mechanism is still unknown [86]. In addition to DDI2, the Ca^2+^-regulatory calpains act as a class of limited proteases that may contribute to the selective proteolytic processing of Nrf1 [87]. However, calpain inhibitors exhibited different effects on Nrf1 transactivation activity, which was observed in varying experimental settings [87,88]. Although most of the ER-resident transcription factors exhibit a membrane topology that localizes the domains required for their transcription activity into the cytosol, the large part of the polypeptide in Nrf1 is located in the ER lumen. The activation mechanisms of other ER-resident transcription factors, such as sterol regulatory element-binding protein (SREBP), activating transcription factor 6 (ATF6), OASIS, and cyclic AMP-responsive element-binding protein H (CREBH), have been well studied to date. All these proteins transit to the Golgi apparatus and are cleaved by site-1 protease (S1P) and site-2 protease (S2P), and are then released from the membranes in order to become activated [89,90]. However, Nrf1 processing occurs in the absence of S1P, S2P, ER membrane-resident rhomboid proteases, or the proteasome. Notably, it was recently shown that Nrf1 is able to enter the nucleus without cleavage [85]. Therefore, the Nrf1 activation mechanism was considered to be resulting from a non-canonical pathway; as such, its precise mechanism remains unknown (Figure 4).

DDI2 was identified as a Nrf1 nuclear translocation regulator by an image-based screening of mammalian cells [91]. By genetic screening using *C. elegans*, ddi1 (*C. elegans* ortholog of DDI2) mutants were identified as having suppressed SKN-1-dependent induction of proteasome gene expression [92]. DDI family proteins consist of a retroviral protease-like (RVP) domain that exhibits a structure closely resembling that of the HIV protease-1, suggesting that DDI proteins are aspartic proteases. In fact, a mutation in the protease active site of DDI2 decreased the production of the ≈110 kDa processed form of Nrf1, whereas the ≈120 kDa full-length form of Nrf1 was accumulated. Additionally, the bounce-back response of proteasome expression against proteasome inhibition was remarkably suppressed by DDI2 depletion and in protease-dead DDI2 mutants in both *C. elegans* and mammals. This indicates that the activation mechanism of Nrf1/SKN-1 by the DDI proteins is widely conserved in multicellular animals.

Studies on yeast Ddi1 (yeast ortholog of DDI2) provided evidence for its proteolytic activity, which was found to be required for sufficient checkpoint regulation, and it also contributes to DNA replication stress response and to DNA-protein crosslink repair; however, very little is known regarding the physiological roles of DDI proteins in mammals [93,94,95]. Yeast Ddi1 contains UBL at the N-terminus and a UBA at the C-terminus. UBL-UBA-containing proteins such as Rad23 and Dsk2 are shuttle factors that interact with ubiquitinated substrates and proteasome subunits through their UBA and UBL domains for degradation. Despite having a similar domain composition, yeast Ddi1 was found to exhibit weaker binding affinity towards ubiquitin chains when compared with those of Rad23 and Dsk2, and its function as a shuttle factor remains unestablished. Moreover, the UBA domain of human DDI2 is substituted in a ubiquitin-interacting motif that weakly binds to ubiquitin [96]. Furthermore, the UBL domain of yeast Ddi1 proteins exhibits an unconventional feature when compared to ubiquitin or other UBL proteins. In ubiquitin and ordinary UBL domains, the hydrophobic patch, which is the interaction site of binding partners, is surrounded by positively charged side chains. However, the hydrophobic patch of the yeast Ddi1 UBL domain is surrounded by negatively charged side chains, suggesting that the binding partners of the yeast Ddi1 UBL domain differ from those of ubiquitin and other UBL domains [97]. Indeed, it was demonstrated that the yeast Ddi1 UBL is an atypical UBL domain that binds to ubiquitin [98].

The UBL domain of human DDI2 is involved in Nrf1 processing [91], probably through its interaction with ubiquitin chains. Consistent with this, blocking ubiquitin chain formation using an E1 inhibitor causes the accumulation of full-length Nrf1 even when DDI2 is present [86]. Further analysis is required to reveal the mechanism by which DDI2 recognizes the substrate and gets activated because Nrf1 processing by the DDI proteins has not been observed in vitro. These studies contribute to the elucidation of the detailed molecular mechanisms behind proteasome upregulation.

### 4.3. Post-Translational Modification Regulates Nrf1

Nrf1 transcription activity is regulated both positively and negatively by phosphorylation and glycosylation.

#### 4.3.1. Glycosylation

N-linked glycosylation and subsequent deglycosylation steps have critical roles in Nrf1 transcription activity. Glycosylation occurs in its Asn/Ser/Thr-rich (NST) domain that consists of seven Asn-X-Ser/Thr consensus motifs for glycosylation. NGLY1 is thought to be responsible for deglycosylating misfolded proteins prior to cytosolic degradation [99,100]. NGLY1 catalyzes cleavage of the amide bond between the proximal GlcNAc and the asparagine of the glycoprotein, resulting in a deglycosylated peptide and a free, intact N-glycan. The deglycosylated, misfolded protein is then degraded by the proteasome whereas the free glycan is recycled in the cell. It has been shown that NGLY1 knockdown causes a reduction of UDP-GlcNAc levels without ER stress, and GlcNAc supplementation rescues the lethal phenotype exhibited by the NGLY1 knockdown without global transcription changes in *Drosophila* [101]. These results imply that NGLY1 may function in an extremely specific target of ERAD and may not be required for global ERAD to clear misfolded proteins. Furthermore, genetic screening in nematodes demonstrated the function of N-glycanase 1 (*png-1*; *C. elegans* ortholog of human NGLY1) which is involved in the bounce-back response of proteasome gene expression. Recently, it was reported that NGLY1 edits the sequence of SKN-1A by converting its glycosylated Asn (-N-S/T-) into acidic Asp (-D-S/T-) residues. This editing is required for DDI-1-mediated processing of SKN-1A and the expression of proteasome genes in defending against proteotoxic stress in *C. elegans* [102]. Accordingly, human NGLY1 is required for Nrf1 transcription activity as it promotes Nrf1 processing and nuclear translocation [84]. Glycosylation-defective Nrf1, in which all the Asns are replaced with Glns, exhibits a decreased transcription activity, whereas the Nrf1 mutant that mimics the deglycosylated state by replacing all the Asns with Asps exhibits an enhanced transcription activity [83]. These data indicate that N-linked glycosylation and subsequent deglycosylation steps have critical roles in Nrf1 function.

In addition to the effects of N-glycosylation on Nrf1, O-GlcNAcylation of Nrf1 by OGT is also essential for Nrf1 stabilization and for the bounce-back response [103,104]. OGT interacts with Nrf1 and modifies Nrf1 with an O-GlcNAc, which then attenuates Nrf1 ubiquitination and increases Nrf1 stability and transcription activity prior to degradation. In contrast, OGT is also known to be involved in the negative regulation of Nrf1 activity. In Nrf1, the serine/threonine-rich region in the proline-glutamate-serine-threonine-rich sequence 2 (PEST2) of Nrf1 is involved in the O-linked glycosylation that downregulates Nrf1 stability and transcription activity [105]. Thus, the dual role of O-linked glycosylation might be involved in regulating Nrf1 activity in response to various cellular conditions.

#### 4.3.2. Phosphorylation

Nrf1 activity is increased by treatment with okadaic acid, which is a phosphatase inhibitor, and is suppressed by the protein kinase C (PKC) inhibitor staurosporine. This suggests that phosphorylation has a positive effect on Nrf1 activity [106]; however, there is no evidence that indicates the direct phosphorylation of Nrf1 by PKC. In contrast, casein kinase 2 (CK2) and glycogen synthase kinase 3 (GSK3β) directly phosphorylate Nrf1 to suppress its activity. CK2 interacts with Nrf1 and phosphorylates the Ser497 residue, whereas CK2 knockdown enhances Nrf1 recruitment to the AREs of the promoter regions of the proteasome subunit genes to upregulate their expression [107]. GSK3β binds to Nrf1 and phosphorylates the Ser350 residue. Treatment with a GSK3β inhibitor or the GSK3β S350A mutant stabilizes Nrf1, as the phosphorylation of Ser350 by GSK3β is required for F-box/WD repeat-containing protein 7 (FBW7)-dependent Nrf1 degradation. Additionally, the expression of Nrf1 S350A mutant ameliorated stress-induced apoptosis in neuronal cells, showing the importance of GSK3β-mediated regulation of Nrf1 activity [108].

## 5. Diverse Function and Regulation of Nrf1

In addition to controlling the expression of proteasome subunit genes, Nrf1 also controls the expression of Herpud1 and p97/VCP, which are components of the ERAD pathway [86,109]. Nrf1 was found to be necessary for Herpud1 basal expression and induction under ER stress in both mouse and human cells. SKN-1 directly activates the expression of many of the core regulators that sense ER stress and control downstream pathways that are important for ER stress resistance [110]. SKN-1 also controls genes that encode chaperones; lysosomal proteases; autophagy proteins; and proteins involved with carbohydrate and amino metabolism, mitochondrial biogenesis, lipid metabolism, and glucose homeostasis, suggesting that it may promote proteostasis at many levels [111,112,113]. Nrf1 has also been reported to play an important role in DNA repair in response to UVB irradiation by regulating the expression of xeroderma pigmentosum C (XPC) [114]. Notably, Nrf1 regulates the expression of USP9X, which is known to be dysfunctional in PD and Lewy body disease, and is responsible for governing neural homeostasis through binding on its consensus ARE sequence [115]. Interestingly, a single nucleotide polymorphism rs667897A found at the membrane-spanning 4-domains subfamily A (MS4A) locus is identified as one of the most important loci relevant to AD, as it creates an additional ARE site that yields in an unexpected linkage of the MS4A6A expression to Nrf1 and Nrf2 [116]. Moreover, the NGLY1–Nrf1 pathway has been revealed to exert an additional novel function in mitochondrial homeostasis and inflammation pathogenesis [117]. Thus, Nrf1 and SKN-1 play roles in regulating numerous biological pathways, although the exact mechanism of their control is still unknown [118].

Because studies on the activation of Nrf1 have been largely restricted to its response to proteasome inhibitors, the precise molecular mechanism behind the nuclear transport and activation of Nrf1 is not entirely clear. A recent study in *C. elegans* revealed that activation of proteasome subunit expression by SKN-1A is triggered by misfolded endogenous proteins and the human amyloid beta peptide, even though proteasome function is not impaired, suggesting that SKN-1A activation is a general protective mechanism against defective proteostasis [58]. Indeed, pharmacological activation of Nrf1 showed a protective role in a mouse model of one age-dependent spinal and bulbar muscular atrophy [119].

Recently, the TIP60 chromatin regulatory complex was shown to be necessary for the Nrf1-dependent transcription of proteasome subunit genes; however, the exact nature of this requirement remains to be uncovered [120].

The proteasome actively contributes to ER quality control through the ERAD [121]. Thus, proteasome inhibition increases the levels of unfolded proteins in the ER, a condition referred to as ER stress. Failure to degrade misfolded proteins in the ER triggers the UPR, which consists of the activation of ER-resident membrane proteins that are capable of sensing perturbed protein homeostasis in the ER. Thus, it is reasonable to use Nrf1 as a monitor of proteostasis because Nrf1 is prone to forming aggregates in the cytoplasm of cells under proteotoxic stress [122]. UPR activation following proteasome inhibition is particularly prominent in secreting cells such as pancreatic β-cells [123]. Unlike the UPR mediators IRE1, ATF6, and PERK, Nrf1 does not constitutively bind to Bip and does not respond to perturbed ER protein-folding homeostasis. In contrast, typical inducers of Nrf1 include proteasome inhibitors, ROS [124], and the presence of excess cholesterol [125]. SKN-1 directly activates expression of UPR genes. On the other hand, UPR transcription factors XBP-1 and ATF6 regulate UPR target genes and also skn-1 expression in *C. elegans* [110].

In *C. elegans*, the ablation of germ-like stem cells causes SKN-1 activation, thereby increasing proteasome activity, stress resistance, and lifespan [126]. Loss-of-function skn-1 mutants have a shortened lifespan, and although the high-level transgenic overexpression of SKN-1 is harmful, lifespan is extended significantly by more modest levels of SKN-1 overexpression [127]. Overexpression of CncC also increases lifespan in *D. melanogaster* [128,129]. Nrf1 knockout leads to embryonic lethality in mice, and fibroblasts derived from Nrf1 knockout embryos have an increased sensitivity to oxidative stress-induced cytotoxicity. Although the basal expression of proteasome genes did not appear to be significantly affected, the bounce-back response against proteasome inhibitors was impaired in Nrf1-deficient cells [16,17]. In contrast, organ-specific knockout of Nrf1 in neurons or in hepatocytes showed an accumulation of ubiquitin-conjugated proteins accompanied with increased oxidative stress and ER stress and decreased expressions of proteasome subunit genes and various *Gst* genes [130,131,132]. Loss of Nrf1 function shows an increase in micronuclei formation and chromosomal instability and also directly promotes chromosome mis-segregation, which then contributes to tumorigenesis [133]. It was also found that osteoblast-specific Nrf1 null mice exhibit a reduction in peak bone mass, bone size, trabecular number, and mechanical strength. Nrf1 knockdown cells induce *HK1* mRNA expression, and HK1 protein was found to be highly increased in the mitochondrial fraction [134]. Transgenic mice with Nrf1 overexpression are characterized by weight loss and protection from diet-induced obesity. In addition, Nrf1 transgenic mice developed insulin resistance through the suppression of insulin signaling via AKT activation in liver and skeletal muscle [135], implying that the efficacy of Nrf1 activation in increasing lifespan is not obvious in mammals. These results indicate that Nrf1 plays an essential role in systemic metabolic homeostasis.

## 6. mTORC1-SREBP Pathway Regulate Nrf1 Activity

In addition to stress-related stimuli, mTORC1-mediated SREBP activity is known to activate Nrf1 expression [136]. mTORC1 promotes cell growth and also activates SREBP, which regulates de novo lipid synthesis [137]. mTORC1-mediated activation of SREBP directly induces Nrf1 expression by four consensus sterol regulatory elements in proximity with the predicted transcriptional start site of the *Nfe2l1* gene, which encodes Nrf1 [138]. Treatment of rapamycin, an mTORC1 inhibitor, decreases the expression of proteasome in the mouse liver [139,140]. Thus, mTORC1 activation leads to increased expression of proteasome through Nrf1 activation in cells and tissues.

mTORC1 also plays a role in the prevention of ER stress through the activation of SREBP and de novo lipid synthesis. Liver-specific deletion of Nrf1 in mice results in hepatic lipid accumulation by disruption of lipid homeostasis, suggesting that Nrf1 controls the lipid metabolism by expression of a subset of enzymes [131,141]. Of note, cold adaptation induces Nrf1 to increase of proteasome activity in brown adipose tissue (BAT). Under thermogenic conditions, deletion of Nrf1 in brown adipocyte results in ER stress, tissue inflammation, impaired mitochondrial dysfunction, and whitening of the BAT [142]. On the basis of this fact, Nrf1-mediated proteasome regulation is considered to be important for BAT function to adapt to either cold or obesity. Excess dietary cholesterol and circulating cholesterol is known as pathogenic in fatty liver, diabetes, atherosclerosis, and neurodegenerative diseases. It has also been shown that Nrf1 directly binds to and senses the hydroxyl group of cholesterol at the ER via cholesterol recognition amino acid consensus domain, and that cholesterol activates Nrf1 to trigger immunometabolic responses for supporting cholesterol homeostasis through regulating Nrf1 cleavage, degradation, and nuclear translocation [125].

## 7. Relationships of Other Nrf Family Transcription Factors

To date, Nrf2 has been assumed to act as a proteasome subunit gene transcription factor until further details on the molecular function of Nrf1 are revealed. In addition, a recent study suggests that the ER-resident Nrf3 has the potential to act with an activity overlapping that of Nrf1, even though the function of Nrf3 is tissue-specific.

### 7.1. Nrf2

Nrf2, which is widely accepted to be a master regulator of adaptive responses to oxidative stress and electrophiles, contains the same consensus binding sequence as Nrf1 [143,144]. Interestingly, ChIP-seq data demonstrated that the target genes of Nrf1 and Nrf2 are slightly overlapping, and that Nrf1, but not Nrf2, predominantly regulates proteasome gene expression. This research shows that Nrf1 showed a slight preference for AREs flanked by AT-rich sequences, whereas Nrf2 showed a slight preference for AREs flanked by GC-rich sequences [145]. Nrf2 is a cytosolic protein and is known to be constantly degraded through cytosolic E3 such as Keap1/Cul3 and SCF-βTrCP complexes in a redox-dependent fashion [146]. Nrf2 has also been known to activate the expression of proteasome subunits upon exposure to oxidative stress, implying that it supports the degradation of proteins damaged by reactive oxygen species [147]. Similar to Rpn4 elevation that enhances replicative lifespan in yeast, Nrf2 activation induces proteasome activity and leads to lifespan extension of human fibroblasts [43,148]. In addition, Nrf2 selectively interacts with p53 gain-of-function mutants, increasing the binding capacity of Nrf2 to proteasome gene promoters in several kinds of cancer cells [149]. This suggests that association with other factors plays an important role in determining the binding specificities of Nrf1 and Nrf2, but these precise mechanisms have yet to be elucidated.

Pharmacologically, tunicamycin only increases Nrf1 protein levels, and sulforaphane only increases Nrf2 protein levels. Moreover, the Nrf1 activity of proteasome induction was shown to be repressed by tunicamycin [150,151]. Nrf1 is required for the transcription of most of UPR-target genes, such as *Chop*, *IRE1*, and *ATF6* genes against tunicamycin, although Nrf2 is also partially involved in this response [152]. In contrast, under ER stress, Nrf2, phosphorylated by PERK, dissociates from Keap1 and then translocates into the nucleus, leading to the activation of ARE genes [153]. Collectively, these demonstrate that the Nrf2-mediated expression of ARE genes is also activated in UPR signaling to ER stress. However, ER stress signaling to activate UPR in the steatotic hepatocytes of a homozygous knockout of Nrf1^-/-^ (but not Nrf2^-/-^) revealed that Nrf1 is necessary for cellular homeostasis and organ integrity, and that it cannot be replaced by Nrf2 [154,155,156].

### 7.2. Nrf3

Nrf3 is controlled in part by a PEST degron sequence and has three isoforms with varying subcellular localizations, including isoform “A” in the ER, isoform “B” in the cytosol, and isoform “C” in the nucleus [157]. Similar to Nrf1, Nrf3 is targeted to the ER and constantly degraded by the ERAD [158,159]. In the nucleus, Nrf3 is also subjected to ubiquitination in a β-TRCP- or GSK3β-dependent manner by Fbw7, which is an adaptor of Cul1-based E3 [160]. Proteasome inhibition activates Nrf3, and DDI2 is required for Nrf3 nuclear translation, although there is still no experimental evidence supporting the idea that DDI2 directly cleaves Nrf3 [161]. A recent study showed that Nrf3 enhances 20S proteasome assembly by directly inducing the gene expression of the 20S proteasome chaperone POMP in cancer cells. Then, ubiquitin-independent degradation of the tumor suppressor p53 and Rb proteins by 20S proteasomes contribute to colorectal cancer development [65]. Nrf3 directly augments *CPEB3* gene expression, and induced CPEB3 represses Nrf1 translation through a cytoplasmic polyadenylation element in the 3′-UTR of the *Nrf1* gene. Recent ChIP-seq data demonstrated that Nrf1 and Nrf3 peaks show relatively similar distributions, suggesting that Nrf1 and Nrf3 act complementarily [145]. In contrast to Nrf1, Nrf3-deficient mice show no apparent abnormalities under physiological conditions. Thus, less is known about Nrf3 function and regulation.

## 8. Nrf1 Regulation Machinery as a Potential Therapeutic Target

Multiple pieces of evidence have unveiled the association between proteasome function and various diseases such as cancer, neurodegeneration, autoinflammation, and autoimmunity. Proteasome inhibitors have been demonstrated to exhibit remarkable effects in the treatment of multiple myeloma and other malignant neoplasms, while the emergence of drug resistance against proteasome inhibitors is a major concern during cancer treatment. Combating these resistance mechanisms can serve as a potentially useful strategy for making proteasome inhibitors more effective for treating cancers and solid tumors. However, there have been multiple observed mechanisms of resistance to proteasome inhibitors, such as mutations in *PSMB5* (a target subunit of the proteasome inhibitor bortezomib), aberrant expression of UPS pathway components, and induction of drug efflux from cells [162]. The bounce-back response mediated by Nrf1 activation is one of the causes of resistance to proteasome inhibitors. Indeed, Nrf1, DDI2, and NGLY1 exhibit a correlated essentiality for cell survival in several acute myeloid leukemia (AML) cell lines [163]. In this regard, Nrf1, as well as the molecules involved in the processing, glycosylation, phosphorylation, and degradation of Nrf1, are promising, as they act as potentially “druggable” candidates for combination therapies with proteasome inhibitors. Structural studies have identified an RVP domain in DDI2 that is similar to the HIV-1 protease (HIVp), which is a therapeutic target for HIV [96]. It has been reported that HIV inhibitors, including ritonavir, nelfinavir, and saquinavir, attenuate proteasome activity and inhibit cancer cell growth in vitro [164,165]. DDI2 and NGLY1 knockdown potentiated sensitivity against a proteasome inhibitor. It has also been shown that NGLY1 inhibitor leads the enhancement of cytotoxicity of proteasome inhibitor. Additionally, a novel NGLY1 inhibitor, WRR139, synergistically promotes cytotoxicity in the presence of a proteasome inhibitor by disrupting the processing, localization, and activation of Nrf1 [84]. Conversely, activation of Nrf1 induced by some drugs or chemicals leads to cytoprotection from cell apoptosis and promotes cell viability [119]. Pharmacological Nrf1 activators have been identified by high-throughput chemical screening [166,167]. Rotenone triggers the ER-to-nuclear translocation of Nrf1 and induces cytoprotection by proteasome against rotenone-stimulated cytotoxic stress [124]. Hrd1/synoviolin, ER-resident E3 for ERAD of Nrf1, is known to be involved in ER stress-induced cell death and is significantly reduced in the cerebral cortex of patients with AD [168]. Another study showed that icariin, a type of flavonoid, induces Hrd1/synoviolin expression through Nrf1-mediated transcription in pheochromocytoma Pi12 cells [169]. ASC-JM17, an orally bioavailable curcumin analog, mitigates polyglutamine toxicity by inducing the antioxidant response and proteasome expression through Nrf1 activation [119]. Inorganic arsenic and t-butyl hydroquinone (TBHQ), which are potent oxidative stress inducers, lead to Nrf1 accumulation in the nucleus in a similar manner to tunicamycin treatment [83,170]. These Nrf1-specific activators or inhibitors should contribute to determine the physiological importance of Nrf1 activation or to reveal molecular mechanisms of Nrf1 regulation. Thus, approaches in exploring the mechanisms behind Nrf1 activation and DDI2 cleavage have important applications.

## 9. Concluding Remarks and Perspectives

Recent studies have unveiled the regulatory mechanisms of Nrf1 activation; however, many questions still remain unsolved. Thus far, the mechanism of DDI2 activation is unclear because the protease activity of purified recombinant DDI2 has not yet been confirmed in vitro. A recent study has shown that yeast Ddi1 cleaves substrate proteins in vitro only when they are tagged with long ubiquitin chains (approximately longer than eight ubiquitins) [171]. It has also been shown that Ddi1 requires the UBL domain, which mediates the high-affinity interaction with the polyubiquitin chain for their activity. Therefore, further studies regarding the regulatory mechanisms behind proteasome expression are essential to provide new strategies that would improve healthy aging and treat various diseases. For example, neurodegenerative diseases such as PD and various prion diseases are characterized by the accumulation of ubiquitinated protein aggregates in neurons, suggesting that there is too little proteasomal activity within these cells [172,173]. Future studies should be conducted to address these questions as they may yield new strategies that are exceedingly valuable in combating these diseases by increasing proteasome synthesis.

## References

[1] Soares T.R., Reis S.D., Pinho B.R., Duchen M.R., Oliveira J.M.A. (2019). Targeting the proteostasis network in Huntington’s disease. Ageing Res. Rev..

[2] Young V.R., Steffee W.P., Pencharz P.B., Winterer J.C., Scrimshaw N.S. (1975). Total human body protein synthesis in relation to protein requirements at various ages. Nature.

[3] Mitch W.E., Goldberg A.L. (1996). Mechanisms of muscle wasting—The role of the Ubiquitin–Proteasome pathway. N. Engl. J. Med..

[4] Hershko A., Aaron C. (1998). The Ubiquitin System. Annu. Rev. Biochem..

[5] Baumeister W., Walz J., Zühl F., Seemüller E. (1998). The proteasome: Paradigm of a self-compartmentalizing protease. Cell.

[6] Varshavsky A. (2005). Regulated protein degradation. Trends Biochem. Sci..

[7] Ciechanover A. (2012). Intracellular protein degradation: From a vague idea thru the lysosome and the ubiquitin-proteasome system and onto human diseases and drug targeting. Biochim. Biophys. Acta Proteins Proteomics.

[8] Tanaka K. (2009). The proteasome: Overview of structure and functions. Proc. Jpn. Acad. Ser. B Phys. Biol. Sci..

[9] Sun Z., Brodsky J.L. (2019). Protein quality control in the secretory pathway. J. Cell Biol..

[10] Berner N., Reutter K.-R., Wolf D.H. (2018). Protein quality control of the endoplasmic reticulum and ubiquitin–proteasome-triggered degradation of aberrant proteins: Yeast pioneers the path. Annu. Rev. Biochem..

[11] Ariyasu D., Yoshida H., Hasegawa Y. (2017). Endoplasmic reticulum (Er) stress and endocrine disorders. Int. J. Mol. Sci..

[12] Hwang J., Qi L. (2018). Quality control in the endoplasmic reticulum: Crosstalk between ERAD and UPR pathways. Trends Biochem. Sci..

[13] Rubinsztein D.C. (2006). The roles of intracellular protein-degradation pathways in neurodegeneration. Nature.

[14] Kumatori A., Tanaka K., Inamura N., Sone S., Ogura T., Matsumoto T., Tachikawa T., Shin S., Ichihara A. (1990). Abnormally high expression of proteasomes in human leukemic cells. Proc. Natl. Acad. Sci. USA.

[15] Chen L., Madura K. (2005). Increased proteasome activity, ubiquitin-conjugating enzymes, and eEF1A translation factor detected in breast cancer tissue. Cancer Res..

[16] Steffen J., Seeger M., Koch A., Krüger E. (2010). Proteasomal degradation is transcriptionally controlled by TCF11 via an ERAD—Dependent feedback loop. Mol. Cell.

[17] Radhakrishnan S.K., Lee C.S., Young P., Beskow A., Chan J.Y., Deshaies R.J. (2010). Transcription factor Nrf1 mediates the proteasome recovery pathway after proteasome inhibition in mammalian cells. Mol. Cell.

[18] Chen Y., Zhang Y., Yin Y., Gao G., Li S., Jiang Y., Gu X., Luo J. (2005). SPD—A web-based secreted protein database. Nucleic Acids Res..

[19] Choi J., Park J., Kim D., Jung K., Kang S., Lee Y.H. (2010). Fungal secretome database: Integrated platform for annotation of fungal secretomes. BMC Genom..

[20] Mehrtash A.B., Hochstrasser M. (2019). Ubiquitin—Dependent protein degradation at the endoplasmic reticulum and nuclear envelope. Semin. Cell Dev. Biol..

[21] Qi L., Tsai B., Arvan P. (2017). New insights into the physiological role of endoplasmic reticulum—Associated degradation. Trends Cell Biol..

[22] Ward C.L., Kopito R.R. (1994). Intracellular turnover of cystic fibrosis transmembrane conductance regulator. Inefficient processing and rapid degradation of wild-type and mutant proteins. J. Biol. Chem..

[23] Lukacs G.L., Mohamed A., Kartner N., Chang X.B., Riordan J.R., Grinstein S. (1994). Conformational maturation of CFTR but not its mutant counterpart (delta F508) occurs in the endoplasmic reticulum and requires ATP. EMBO J..

[24] Shi G., Somlo D., Kim G.H., Prescianotto-Baschong C., Sun S., Beuret N., Long Q., Rutishauser J., Arvan P., Spiess M. (2017). ER–Associated degradation is required for vasopressin prohormone processing and systemic water homeostasis. J. Clin. Investig..

[25] Sun S., Shi G., Han X., Francisco A.B., Ji Y., Mendonça N., Liu X., Locasale J.W., Simpson K.W., Duhamel G.E. (2014). Sel1L is indispensable for mammalian endoplasmic reticulum—Associated degradation, endoplasmic reticulum homeostasis, and survival. Proc. Natl. Acad. Sci. USA.

[26] Sun S., Lourie R., Cohen S.B., Ji Y., Goodrich J.K., Poole A.C., Ley R.E., Denkers E.Y., Mcguckin M.A., Long Q. (2016). Epithelial sel1L is required for the maintenance of intestinal homeostasis. Mol. Biol. Cell.

[27] Sha H., Sun S., Francisco A.B., Ehrhardt N., Xue Z., Liu L., Lawrence P., Mattijssen F., Guber R.D., Panhwar M.S. (2014). The ER-associated degradation adaptor protein sel1l regulates LPL secretion and lipid metabolism. Cell Metab..

[28] Wangeline M.A., Vashistha N., Hampton R.Y. (2017). Proteostatic tactics in the strategy of sterol regulation. Annu. Rev. Cell Dev. Biol..

[29] Wojcikiewicz R.J.H., Pearce M.M.P., Sliter D.A., Wang Y. (2009). When worlds collide: IP3 receptors and the ERAD pathway. Cell Calcium.

[30] Enenkel C., Lehmann A., Kloetzel P.M. (1998). Subcellular distribution of proteasomes implicates a major location of protein degradation in the nuclear envelope-ER network in yeast. EMBO J..

[31] Palmer A., Rivett A.J., Thomson S., Hendil K.B., Butcher G.W., Fuertes G., Knecht E. (1996). Subpopulations of proteasomes in rat liver nuclei, microsomes and cytosol. Biochem. J..

[32] Albert S., Wietrzynski W., Lee C.W., Schaffer M., Beck F., Schuller J.M., Salomé P.A., Plitzko J.M., Baumeister W., Engel B.D. (2020). Direct visualization of degradation microcompartments at the ER membrane. Proc. Natl. Acad. Sci. USA.

[33] Finley D., Chen X., Walters K.J. (2016). Gates, channels, and switches: Elements of the proteasome machine. Trends Biochem. Sci..

[34] Śledź P., Baumeister W. (2016). Structure—Driven developments of 26S proteasome inhibitors. Annu. Rev. Pharmacol. Toxicol..

[35] Murata S., Takahama Y., Kasahara M., Tanaka K. (2018). The immunoproteasome and thymoproteasome: Functions, evolution and human disease. Nat. Immunol..

[36] Bard J.A.M., Goodall E.A., Greene E.R., Jonsson E., Dong K.C., Martin A. (2018). Structure and function of the 26S proteasome. Annu. Rev. Biochem..

[37] Bhattacharyya S., Yu H., Mim C., Matouschek A. (2014). regulated protein turnover: Snapshots of the proteasome in action. Nat. Rev. Mol. Cell Biol..

[38] Grice G.L., Nathan J.A. (2016). The recognition of ubiquitinated proteins by the proteasome. Cell. Mol. Life Sci..

[39] Trempe J.-F. (2011). Reading the ubiquitin postal code. Curr. Opin. Struct. Biol..

[40] Sahara K., Kogleck L., Yashiroda H., Murata S. (2014). The mechanism for molecular assembly of the proteasome. Adv. Biol. Regul..

[41] Rousseau A., Bertolotti A. (2018). Regulation of proteasome assembly and activity in health and disease. Nat. Rev. Mol. Cell Biol..

[42] Albornoz N., Bustamante H., Soza A., Burgos P. (2019). Cellular responses to proteasome inhibition: Molecular mechanisms and beyond. Int. J. Mol. Sci..

[43] Kruegel U., Robison B., Dange T., Kahlert G., Delaney J.R., Kotireddy S., Tsuchiya M., Tsuchiyama S., Murakami C.J., Schleit J. (2011). Elevated proteasome capacity extends replicative lifespan in saccharomyces cerevisiae. PLoS Genet..

[44] Meiners S., Heyken D., Weller A., Ludwig A., Stangl K., Kloetzel P.M., Krüger E. (2003). Inhibition of proteasome activity induces concerted expression of proteasome genes and de novo formation of mammalian proteasomes. J. Biol. Chem..

[45] Radhakrishnan S.K., den Besten W., Deshaies R.J. (2014). p97-dependent retrotranslocation and proteolytic processing govern formation of active Nrf1 upon proteasome inhibition. eLife.

[46] Tonoki A., Kuranaga E., Tomioka T., Hamazaki J., Murata S., Tanaka K., Miura M. (2009). Genetic evidence linking age—Dependent attenuation of the 26S proteasome with the aging process. Mol. Cell. Biol..

[47] Vilchez D., Morantte I., Liu Z., Douglas P.M., Merkwirth C., Rodrigues A.P.C., Manning G., Dillin A. (2012). RPN-6 determines C. elegans longevity under proteotoxic stress conditions. Nature.

[48] Chondrogianni N., Tzavelas C., Pemberton A.J., Nezis I.P., Rivett A.J., Gonos E.S. (2005). Overexpression of proteasome β5 subunit increases the amount of assembled proteasome and confers ameliorated response to oxidative stress and higher survival rates. J. Biol. Chem..

[49] Munkácsy E., Chocron E.S., Quintanilla L., Gendron C.M., Pletcher S.D., Pickering A.M. (2019). Neuronal-specific proteasome augmentation via Prosβ5 overexpression extends lifespan and reduces age-related cognitive decline. Aging Cell.

[50] Howell L.A., Peterson A.K., Tomko R.J. (2019). Proteasome subunit α1 overexpression preferentially drives canonical proteasome biogenesis and enhances stress tolerance in yeast. Sci. Rep..

[51] Nguyen N.N., Rana A., Goldman C., Moore R., Tai J., Hong Y., Shen J., Walker D.W., Hur J.H. (2019). Proteasome β5 subunit overexpression improves proteostasis during aging and extends lifespan in Drosophila melanogaster. Sci. Rep..

[52] Marshall R.S., Vierstra R.D. (2019). Dynamic regulation of the 26S proteasome: From synthesis to degradation. Front. Mol. Biosci..

[53] Shirozu R., Yashiroda H., Murata S. (2015). Identification of minimum Rpn4-responsive elements in genes related to proteasome functions. FEBS Lett..

[54] Xie Y., Varshavsky A. (2001). RPN4 is a ligand, substrate, and transcriptional regulator of the 26S proteasome: A negative feedback circuit. Proc. Natl. Acad. Sci. USA.

[55] Wang X., Xu H., Ju D., Xie Y. (2008). Disruption of Rpn4-induced proteasome expression in Saccharomyces cerevisiae reduces cell viability under stressed conditions. Genetics.

[56] Ma M., Liu Z.L. (2010). Comparative transcriptome profiling analyses during the lag phase uncover YAP1, PDR1, PDR3, RPN4, and HSF1 as key regulatory genes in genomic adaptation to the lignocellulose derived inhibitor HMF for Saccharomyces cerevisiae. BMC Genomics.

[57] Blackwell T.K., Steinbaugh M.J., Hourihan J.M., Ewald C.Y., Isik M. (2015). SKN-1/Nrf, stress responses, and aging in Caenorhabditis elegans. Free Radic. Biol. Med..

[58] Lehrbach N.J., Ruvkun G. (2019). Endoplasmic reticulum-associated SKN-1A/Nrf1 mediates a cytoplasmic unfolded protein response and promotes longevity. eLife.

[59] Qu M., Li Y., Wu Q., Xia Y., Wang D. (2017). Neuronal ERK signaling in response to graphene oxide in nematode Caenorhabditis elegans. Nanotoxicology.

[60] An J.H., Blackwell T.K. (2003). SKN-1 links C. elegans mesendodermal specification to a conserved oxidative stress response. Genes Dev..

[61] Grimberg K.B., Beskow A., Lundin D., Davis M.M., Young P. (2011). Basic Leucine Zipper Protein Cnc-C Is a Substrate and Transcriptional Regulator of the Drosophila 26S Proteasome. Mol. Cell. Biol..

[62] Pitoniak A., Bohmann D. (2015). Mechanisms and functions of Nrf2 signaling in Drosophila. Free Radic. Biol. Med..

[63] Zhang Y., Xiang Y. (2016). Molecular and cellular basis for the unique functioning of Nrf1, an indispensable transcription factor for maintaining cell homoeostasis and organ integrity. Biochem. J..

[64] Fuse Y., Kobayashi M. (2017). Conservation of the Keap1-Nrf2 system: An evolutionary journey through stressful space and time. Molecules.

[65] Waku T., Nakamura N., Koji M., Watanabe H., Katoh H., Tatsumi C., Tamura N., Hatanaka A., Hirose S., Katayama H. (2020). NRF3-POMP-20S proteasome assembly axis promotes cancer development via ubiquitin-independent proteolysis of p53 and Rb. Mol. Cell. Biol..

[66] Wang M., Qiu L., Ru X., Song Y., Zhang Y. (2019). Distinct isoforms of Nrf1 diversely regulate different subsets of its cognate target genes. Sci. Rep..

[67] Xu H., Fu J., Ha S.W., Ju D., Zheng J., Li L., Xie Y. (2012). The CCAAT box-binding transcription factor NF-Y regulates basal expression of human proteasome genes. Biochim. Biophys. Acta Mol. Cell Res..

[68] Vilchez D., Boyer L., Morantte I., Lutz M., Merkwirth C., Joyce D., Spencer B., Page L., Masliah E., Berggren W.T. (2012). Increased proteasome activity in human embryonic stem cells is regulated by PSMD11. Nature.

[69] Vangala J.R., Dudem S., Jain N., Kalivendi S.V. (2014). Regulation of psmb5 protein and β subunits of mammalian proteasome by constitutively activated signal transducer and activator of transcription 3 (stat3): Potential role in bortezomib-mediated anticancer therapy. J. Biol. Chem..

[70] Zhu Y.P., Wang M., Xiang Y., Qiu L., Hu S., Zhang Z., Mattjus P., Zhu X., Zhang Y. (2018). Nach is a novel subgroup at an early evolutionary stage of the CNC-bZIP subfamily transcription factors from the marine bacteria to humans. Int. J. Mol. Sci..

[71] Bugno M., Daniel M., Chepelev N.L., Willmore W.G. (2015). Changing gears in Nrf1 research, from mechanisms of regulation to its role in disease and prevention. Biochim. Biophys. Acta Gene Regul. Mech..

[72] Koizumi S., Hamazaki J., Murata S. (2018). Transcriptional regulation of the 26S proteasome by Nrf1. Proc. Jpn. Acad. Ser. B Phys. Biol. Sci..

[73] Johnsen O., Skammelsrud N., Luna L., Nishizawa M., Prydz H., Kolsto A.B. (1996). Small maf proteins interact with the human transcription factor TCF11/Nrf1/LCR-F1. Nucleic Acids Res..

[74] Bowerman B., Eaton B.A., Priess J.R. (1992). Skn-1, a maternally expressed gene required to specify the fate of ventral blastomeres in the early C. elegans embryo. Cell.

[75] Blackwell T.K., Bowerman B., Priess J.R., Weintraub H. (1994). Formation of a monomeric DNA binding domain by Skn-1 bZIP and homeodomain elements. Science.

[76] Carroll A.S., Gilbert D.E., Liu X., Cheung J.W., Michnowicz J.E., Wagner G., Ellenberger T.E., Blackwell T.K. (1997). SKN-1 domain folding and basic region monomer stabilization upon DNA binding. Genes Dev..

[77] Baird L., Tsujita T., Kobayashi E., Funayama R., Nagashima T., Nakayama K., Yamamoto M. (2016). A Homeostatic shift facilitates endoplasmic reticulum proteostasis through transcriptional integration of proteostatic stress response pathways. Mol. Cell. Biol..

[78] Tsuchiya Y., Morita T., Kim M., Iemura S., Natsume T., Yamamoto M., Kobayashi A. (2011). Dual regulation of the transcriptional activity of Nrf1 by β-TrCP- and Hrd1-dependent degradation mechanisms. Mol. Cell. Biol..

[79] Biswas M., Phan D., Watanabe M., Chan J.Y. (2011). The Fbw7 tumor suppressor regulates nuclear factor E2-related dactor 1 transcription factor turnover through proteasome-mediated proteolysis. J. Biol. Chem..

[80] Fukagai K., Waku T., Chowdhury A.M.M.A., Kubo K., Matsumoto M., Kato H., Natsume T., Tsuruta F., Chiba T., Taniguchi H. (2016). USP15 stabilizes the transcription factor Nrf1 in the nucleus, promoting the proteasome gene expression. Biochem. Biophys. Res. Commun..

[81] Choe K.P., Przybysz A.J., Strange K. (2009). The WD40 Repeat Protein WDR-23 Functions with the CUL4/DDB1 ubiquitin ligase to regulate nuclear abundance and activity of SKN-1 in caenorhabditis elegans. Mol. Cell. Biol..

[82] Zhang Y., Lucocq J.M., Yamamoto M., Hayes J.D. (2007). The NHB1 (N-terminal homology box 1) sequence in transcription factor Nrf1 is required to anchor it to the endoplasmic reticulum and also to enable its asparagine-glycosylation. Biochem. J..

[83] Zhang Y., Lucocq J.M., Hayes J.D. (2009). The Nrf1 CNC/bZIP protein is a nuclear envelope-bound transcription factor that is activated by t-butyl hydroquinone but not by endoplasmic reticulum stressors. Biochem. J..

[84] Tomlin F.M., Gerling-Driessen U.I.M., Liu Y.-C., Flynn R.A., Vangala J.R., Lentz C.S., Clauder-Muenster S., Jakob P., Mueller W.F., Ordoñez-Rueda D. (2017). Inhibition of NGLY1 Inactivates the Transcription factor Nrf1 and potentiates proteasome inhibitor cytotoxicity. ACS Cent. Sci..

[85] Northrop A., Vangala J.R., Feygin A., Radhakrishnan S.K. (2020). Disabling the protease DDI2 attenuates the transcriptional activity of NRF1 and potentiates proteasome inhibitor cytotoxicity. Int. J. Mol. Sci..

[86] Sha Z., Goldberg A.L. (2014). Proteasome-Mediated Processing of Nrf1 Is Essential for Coordinate Induction of All Proteasome Subunits and p97. Curr. Biol..

[87] Nowak K., Taubert R.M., Haberecht S., Venz S., Krüger E. (2018). Inhibition of calpain-1 stabilizes TCF11/Nrf1 but does not affect its activation in response to proteasome inhibition. Biosci. Rep..

[88] Zhang Y., Li S., Xiang Y., Qiu L., Zhao H., Hayes J.D. (2015). The selective post-translational processing of transcription factor Nrf1 yields distinct isoforms that dictate its ability to differentially regulate gene expression. Sci. Rep..

[89] Shao W., Espenshade P.J. (2012). Expanding roles for SREBP in metabolism. Cell Metab..

[90] Stauffer W.T., Arrieta A., Blackwood E.A., Glembotski C.C. (2020). Sledgehammer to scalpel: Broad challenges to the heart and other tissues yield specific cellular responses via transcriptional regulation of the ER-stress master regulator ATF6α. Int. J. Mol. Sci..

[91] Koizumi S., Irie T., Hirayama S., Sakurai Y., Yashiroda H., Naguro I., Ichijo H., Hamazaki J., Murata S. (2016). The aspartyl protease DDI2 activates Nrf1 to compensate for proteasome dysfunction. eLife.

[92] Lehrbach N.J., Ruvkun G. (2016). Proteasome dysfunction triggers activation of SKN-1A/Nrf1 by the aspartic protease DDI-1. eLife.

[93] Mótyán J.A., Miczi M., Tőzsér J. (2020). Dimer interface organization is a main determinant of intermonomeric interactions and correlates with evolutionary relationships of retroviral and retroviral-like ddi1 and ddi2 proteases. Int. J. Mol. Sci..

[94] Serbyn N., Noireterre A., Bagdiul I., Plank M., Michel A.H., Loewith R., Kornmann B., Stutz F. (2020). The aspartic protease Ddi1 contributes to DNA-Protein crosslink repair in yeast. Mol. Cell.

[95] Svoboda M., Konvalinka J., Trempe J.F., Grantz Saskova K. (2019). The yeast proteases Ddi1 and Wss1 are both involved in the DNA replication stress response. DNA Repair (Amst.).

[96] Sivá M., Svoboda M., Veverka V., Trempe J.F., Hofmann K., Kožíšek M., Hexnerová R., Sedlák F., Belza J., Brynda J. (2016). Human DNA-Damage-Inducible 2 Protein Is Structurally and Functionally Distinct from Its Yeast Ortholog. Sci. Rep..

[97] Nowicka U., Zhang D., Walker O., Krutauz D., Castañeda C.A., Chaturvedi A., Chen T.Y., Reis N., Glickman M.H., Fushman D. (2015). DNA-damage-inducible 1 protein (Ddi1) contains an uncharacteristic ubiquitin-like domain that binds ubiquitin. Structure.

[98] Ivantsiv Y., Kaplun L., Tzirkin-Goldin R., Shabek N., Raveh D. (2006). Unique role for the UbL-UbA protein Ddi1 in turnover of SCFUfo1 complexes. Mol. Cell. Biol..

[99] Suzuki T., Huang C., Harada Y., Hosomi A., Masahara-Negishi Y., Seino J., Fujihira H., Funakoshi Y., Suzuki T., Dohmae N. (2015). Endo-β-n-acetylglucosaminidase forms N-GlcNAc protein aggregates during ER-associated degradation in NGLY1-defective cells. Proc. Natl. Acad. Sci. USA.

[100] Suzuki T., Huang C., Fujihira H. (2016). The cytoplasmic peptide: N-glycanase (NGLY1)—Structure, expression and cellular functions. Gene.

[101] Owings K.G., Lowry J.B., Bi Y., Might M., Chow C.Y. (2018). Transcriptome and functional analysis in a Drosophila model of NGLY1 deficiency provides insight into therapeutic approaches. Hum. Mol. Genet..

[102] Lehrbach N.J., Breen P.C., Ruvkun G. (2019). Protein Sequence Editing of SKN-1A/Nrf1 by Peptide:N-Glycanase Controls Proteasome Gene Expression. Cell.

[103] Han J.W., Valdez J.L., Ho D.V., Lee C.S., Kim H.M., Wang X., Huang L., Chan J.Y. (2017). Nuclear factor-erythroid-2 related transcription factor-1 (Nrf1) is regulated by O-GlcNAc transferase. Free Radic. Biol. Med..

[104] Sekine H., Okazaki K., Kato K., Alam M.M., Shima H., Katsuoka F., Tsujita T., Suzuki N., Kobayashi A., Igarashi K. (2018). *O* -GlcNAcylation signal mediates proteasome inhibitor resistance in cancer cells by stabilizing NRF1. Mol. Cell. Biol..

[105] Chen J., Liu X., Lü F., Liu X., Ru Y., Ren Y., Yao L., Zhang Y. (2015). Transcription factor Nrf1 is negatively regulated by its O-GlcNAcylation status. FEBS Lett..

[106] Chepelev N.L., Bennitz J.D., Huang T., McBride S., Willmore W.G. (2011). The Nrf1 CNC-bZIP Protein Is Regulated by the Proteasome and Activated by Hypoxia. PLoS ONE.

[107] Tsuchiya Y., Taniguchi H., Ito Y., Morita T., Karim M.R., Ohtake N., Fukagai K., Ito T., Okamuro S., Iemura S.-I. (2013). The Casein Kinase 2-Nrf1 Axis Controls the Clearance of Ubiquitinated Proteins by Regulating Proteasome Gene Expression. Mol. Cell. Biol..

[108] Biswas M., Kwong E.K., Park E., Nagra P., Chan J.Y. (2013). Glycogen synthase kinase 3 regulates expression of nuclear factor-erythroid-2 related transcription factor-1 (Nrf1) and inhibits pro-survival function of Nrf1. Exp. Cell Res..

[109] Ho D.V., Chan J.Y. (2015). Induction of Herpud1 expression by ER stress is regulated by Nrf1. FEBS Lett..

[110] Glover-Cutter K.M., Lin S., Blackwell T.K. (2013). Integration of the unfolded protein and oxidative stress responses through SKN-1/Nrf. PLoS Genet..

[111] Paek J., Lo J.Y., Narasimhan S.D., Nguyen T.N., Glover-Cutter K., Robida-Stubbs S., Suzuki T., Yamamoto M., Blackwell T.K., Curran S.P. (2012). Mitochondrial SKN-1/Nrf mediates a conserved starvation response. Cell Metab..

[112] Oliveira R.P., Abate J.P., Dilks K., Landis J., Ashraf J., Murphy C.T., Blackwell T.K. (2009). Condition-adapted stress and longevity gene regulation by Caenorhabditis elegans SKN-1/Nrf. Aging Cell.

[113] Pang S., Lynn D.A., Lo J.Y., Paek J., Curran S.P. (2014). SKN-1 and Nrf2 couples proline catabolism with lipid metabolism during nutrient deprivation. Nat. Commun..

[114] Han W., Ming M., Zhao R., Pi J., Wu C., He Y.Y. (2012). Nrf1 CNC-bZIP protein promotes cell survival and nucleotide excision repair through maintaining glutathione homeostasis. J. Biol. Chem..

[115] Taniguchi H., Okamuro S., Koji M., Waku T., Kubo K., Hatanaka A., Sun Y., Chowdhury A.M.M.A., Fukamizu A., Kobayashi A. (2017). Possible roles of the transcription factor Nrf1 (NFE2L1) in neural homeostasis by regulating the gene expression of deubiquitinating enzymes. Biochem. Biophys. Res. Commun..

[116] Lacher S.E., Alazizi A., Wang X., Bell D.A., Pique-Regi R., Luca F., Slattery M. (2018). A hypermorphic antioxidant response element is associated with increased MS4A6A expression and Alzheimer’s disease. Redox Biol..

[117] Yang K., Huang R., Fujihira H., Suzuki T., Yan N. (2018). N-glycanase NGLY1 regulates mitochondrial homeostasis and inflammation through NRF1. J. Exp. Med..

[118] Kim H.M., Han J.W., Chan J.Y. (2016). Nuclear factor Erythroid-2 like 1 (NFE2L1): Structure, function and regulation. Gene.

[119] Bott L.C., Badders N.M., Chen K.L., Harmison G.G., Bautista E., Shih C.C.Y., Katsuno M., Sobue G., Taylor J.P., Dantuma N.P. (2016). A small-molecule Nrf1 and Nrf2 activator mitigates polyglutamine toxicity in spinal and bulbar muscular atrophy. Hum. Mol. Genet..

[120] Vangala J.R., Radhakrishnan S.K. (2019). Nrf1-mediated transcriptional regulation of the proteasome requires a functional TIP60 complex. J. Biol. Chem..

[121] Ebstein F., Poli Harlowe M.C., Studencka-Turski M., Krüger E. (2019). Contribution of the Unfolded Protein Response (UPR) to the Pathogenesis of Proteasome-Associated Autoinflammatory Syndromes (PRAAS). Front. Immunol..

[122] Sha Z., Goldberg A.L. (2016). Reply to Vangala et al.: Complete inhibition of the proteasome reduces new proteasome production by causing Nrf1 aggregation. Curr. Biol..

[123] Kitiphongspattana K., Mathews C.E., Leiter E.H., Gaskins H.R. (2005). Proteasome inhibition alters glucose-stimulated (pro)insulin secretion and turnover in pancreatic β-cells. J. Biol. Chem..

[124] Sotzny F., Schormann E., Kühlewindt I., Koch A., Brehm A., Goldbach-Mansky R., Gilling K.E., Krüger E. (2016). TCF11/Nrf1-Mediated induction of proteasome expression prevents cytotoxicity by rotenone. Antioxid. Redox Signal..

[125] Widenmaier S.B., Snyder N.A., Nguyen T.B., Arduini A., Lee G.Y., Arruda A.P., Saksi J., Bartelt A., Hotamisligil G.S. (2017). NRF1 Is an ER Membrane Sensor that Is Central to Cholesterol Homeostasis. Cell.

[126] Steinbaugh M.J., Narasimhan S.D., Robida-Stubbs S., Moronetti Mazzeo L.E., Dreyfuss J.M., Hourihan J.M., Raghavan P., Operaña T.N., Esmaillie R., Blackwell T.K. (2015). Lipid-mediated regulation of SKN-1/Nrf in response to germ cell absence. eLife.

[127] Tullet J.M.A., Hertweck M., An J.H., Baker J., Hwang J.Y., Liu S., Oliveira R.P., Baumeister R., Blackwell T.K. (2008). Direct inhibition of the longevity-promoting factor SKN-1 by insulin-like signaling in C. elegans. Cell.

[128] Sykiotis G.P., Bohmann D. (2010). Stress-activated cap’n’collar transcription factors in aging and human disease. Sci. Signal..

[129] Sykiotis G.P., Bohmann D. (2008). Keap1/Nrf2 Signaling regulates oxidative stress tolerance and lifespan in drosophila. Dev. Cell.

[130] Lee C.S., Lee C., Hu T., Nguyen J.M., Zhang J., Martin M.V., Vawter M.P., Huang E.J., Chan J.Y. (2011). Loss of nuclear factor E2-related factor 1 in the brain leads to dysregulation of proteasome gene expression and neurodegeneration. Proc. Natl. Acad. Sci. USA.

[131] Lee C.S., Ho D.V., Chan J.Y. (2013). Nuclear factor-erythroid 2-related factor 1 regulates expression of proteasome genes in hepatocytes and protects against endoplasmic reticulum stress and steatosis in mice. FEBS J..

[132] Xu Z., Chen L., Leung L., Yen T.S.B., Lee C., Chan J.Y. (2005). Liver-specific inactivation of the Nrf1 gene in adult mouse leads to nonalcoholic steatohepatitis and hepatic neoplasia. Proc. Natl. Acad. Sci. USA.

[133] Yuan J., Zhang S., Zhang Y. (2018). Nrf1 is paved as a new strategic avenue to prevent and treat cancer, neurodegenerative and other diseases. Toxicol. Appl. Pharmacol..

[134] Fu J., Zheng H., Cui Q., Chen C., Bao S., Sun J., Li L., Yang B., Wang H., Hou Y. (2018). Nfe2l1-silenced insulinoma cells acquire aggressiveness and chemoresistance. Endocr. Relat. Cancer.

[135] Hirotsu Y., Higashi C., Fukutomi T., Katsuoka F., Tsujita T., Yagishita Y., Matsuyama Y., Motohashi H., Uruno A., Yamamoto M. (2014). Transcription factor NF-E2-related factor 1 impairs glucose metabolism in mice. Genes Cells.

[136] Zhang Y., Manning B.D. (2015). mTORC1 signaling activates NRF1 to increase cellular proteasome levels. Cell Cycle.

[137] Horton J.D., Goldstein J.L., Brown M.S. (2002). SREBPs: Activators of the complete program of cholesterol and fatty acid synthesis in the liver. J. Clin. Investig..

[138] Zhang Y., Nicholatos J., Dreier J.R., Ricoult S.J.H., Widenmaier S.B., Hotamisligil G.S., Kwiatkowski D.J., Manning B.D. (2014). Coordinated regulation of protein synthesis and degradation by mTORC1. Nature.

[139] Fok W.C., Chen Y., Bokov A., Zhang Y., Salmon A.B., Diaz V., Javors M., Wood W.H., Zhang Y., Becker K.G. (2014). Mice fed rapamycin have an increase in lifespan associated with major changes in the liver transcriptome. PLoS ONE.

[140] Zhang Y., Bokov A., Gelfond J., Soto V., Ikeno Y., Hubbard G., Diaz V., Sloane L., Maslin K., Treaster S. (2014). Rapamycin extends life and health in C57BL/6 mice. J. Gerontol. Ser. A Biol. Sci. Med. Sci..

[141] Hirotsu Y., Hataya N., Katsuoka F., Yamamoto M. (2012). NF-E2-Related factor 1 (Nrf1) serves as a novel regulator of hepatic lipid metabolism through regulation of the Lipin1 and PGC-1 genes. Mol. Cell. Biol..

[142] Bartelt A., Widenmaier S.B., Schlein C., Johann K., Goncalves R.L.S., Eguchi K., Fischer A.W., Parlakgül G., Snyder N.A., Nguyen T.B. (2018). Brown adipose tissue thermogenic adaptation requires Nrf1-mediated proteasomal activity. Nat. Med..

[143] Higgins L.G., Kelleher M.O., Eggleston I.M., Itoh K., Yamamoto M., Hayes J.D. (2009). Transcription factor Nrf2 mediates an adaptive response to sulforaphane that protects fibroblasts in vitro against the cytotoxic effects of electrophiles, peroxides and redox-cycling agents. Toxicol. Appl. Pharmacol..

[144] Xiao H., Lü F., Stewart D., Zhang Y. (2013). Mechanisms underlying chemopreventive effects of flavonoids via multiple signaling nodes within Nrf2-ARE and AhR-XRE gene regulatory networks. Curr. Chem. Biol..

[145] Liu P., Kerins M.J., Tian W., Neupane D., Zhang D.D., Ooi A. (2019). Differential and overlapping targets of the transcriptional regulators NRF1, NRF2, and NRF3 in human cells. J. Biol. Chem..

[146] Rada P., Rojo A.I., Chowdhry S., McMahon M., Hayes J.D., Cuadrado A. (2011). SCF/ -TrCP Promotes Glycogen Synthase Kinase 3-Dependent degradation of the Nrf2 transcription factor in a Keap1-Independent manner. Mol. Cell. Biol..

[147] Kwak M.-K., Wakabayashi N., Greenlaw J.L., Yamamoto M., Kensler T.W. (2003). Antioxidants enhance mammalian proteasome expression through the Keap1-Nrf2 signaling pathway. Mol. Cell. Biol..

[148] Kapeta S., Chondrogianni N., Gonos E.S. (2010). Nuclear erythroid factor 2-mediated proteasome activation delays senescence in human fibroblasts. J. Biol. Chem..

[149] Walerych D., Lisek K., Sommaggio R., Piazza S., Ciani Y., Dalla E., Rajkowska K., Gaweda-Walerych K., Ingallina E., Tonelli C. (2016). Proteasome machinery is instrumental in a common gain-of-function program of the p53 missense mutants in cancer. Nat. Cell Biol..

[150] Wang W., Chan J.Y. (2006). Nrf1 is targeted to the endoplasmic reticulum membrane by an N-terminal transmembrane domain: Inhibition of nuclear translocation and transacting function. J. Biol. Chem..

[151] Thimmulappa R.K., Mai K.H., Srisuma S., Kensler T.W., Yamamoto M., Biswal S. (2002). Identification of Nrf2-regulated genes induced by the chemopreventive agent sulforaphane by oligonucleotide microarray. Cancer Res..

[152] Zhu Y.P., Zheng Z., Hu S., Ru X., Fan Z., Qiu L., Zhang Y. (2020). Unification of opposites between two antioxidant transcription factors nrf1 and nrf2 in mediating distinct cellular responses to the endoplasmic reticulum stressor tunicamycin. Antioxidants.

[153] Cullinan S.B., Diehl J.A. (2004). PERK—Dependent activation of Nrf2 contributes to redox homeostasis and cell survival following endoplasmic reticulum Stress. J. Biol. Chem..

[154] Chan J.Y., Kwong M., Lu R., Chang J., Wang B., Yen T.S.B., Kan Y.W. (1998). Targeted disruption of the ubiquitous CNC-bZIP transcription factor, Nrf-1, results in anemia and embryonic lethality in mice. EMBO J..

[155] Leung L., Kwong M., Hou S., Lee C., Chan J.Y. (2003). Deficiency of the Nrf1 and Nrf2 transcription factors results in early embryonic lethality and severe oxidative stress. J. Biol. Chem..

[156] Ohtsuji M., Katsuoka F., Kobayashi A., Aburatani H., Hayes J.D., Yamamoto M. (2008). Nrf1 and Nrf2 play distinct roles in activation of antioxidant response element-dependent genes. J. Biol. Chem..

[157] Zhang Y., Kobayashi A., Yamamoto M., Hayes J.D. (2009). The Nrf3 transcription factor is a membrane-bound glycoprotein targeted to the endoplasmic reticulum through its N-terminal homology box 1 sequence. J. Biol. Chem..

[158] Nouhi Z., Chevillard G., Derjuga A., Blank V. (2007). Endoplasmic reticulum association and N-linked glycosylation of the human Nrf3 transcription factor. FEBS Lett..

[159] Chowdhury A.M.M.A., Katoh H., Hatanaka A., Iwanari H., Nakamura N., Hamakubo T., Natsume T., Waku T., Kobayashi A. (2017). Multiple regulatory mechanisms of the biological function of NRF3 (NFE2L3) control cancer cell proliferation. Sci. Rep..

[160] Kannan M.B., Dodard-Friedman I., Blank V. (2015). Stringent control of NFE2L3 (Nuclear Factor, Erythroid 2-Like 3; NRF3) protein degradation by FBW7 (F-box/WD Repeatcontaining–Protein 7) and glycogen synthase kinase 3 (GSK3). J. Biol. Chem..

[161] Kobayashi A., Waku T. (2020). New addiction to the NRF2-related factor NRF3 in cancer cells: Ubiquitin-independent proteolysis through the 20S proteasome. Cancer Sci..

[162] Sherman D.J., Li J. (2020). Proteasome inhibitors: Harnessing proteostasis to combat disease. Molecules.

[163] Wang T., Yu H., Hughes N.W., Liu B., Kendirli A., Klein K., Chen W.W., Lander E.S., Sabatini D.M. (2017). Gene essentiality profiling reveals gene networks and synthetic lethal interactions with oncogenic ras. Cell.

[164] Kraus M., Müller-Ide H., Rückrich T., Bader J., Overkleeft H., Driessen C. (2014). Ritonavir, nelfinavir, saquinavir and lopinavir induce proteotoxic stress in acute myeloid leukemia cells and sensitize them for proteasome inhibitor treatment at low micromolar drug concentrations. Leuk. Res..

[165] Fassmannová D., Sedlák F., Sedláček J., Špička I., Grantz Šašková K. (2020). Nelfinavir Inhibits the TCF11/Nrf1-Mediated proteasome recovery pathway in multiple myeloma. Cancers.

[166] Tsujita T., Baird L., Furusawa Y., Katsuoka F., Hou Y., Gotoh S., Kawaguchi S.I., Yamamoto M. (2015). Discovery of an NRF1-specific inducer from a large-scale chemical library using a direct NRF1-protein monitoring system. Genes Cells.

[167] Iaconelli J., Ibrahim L., Chen E., Hull M., Schultz P.G., Bollong M.J. (2019). Small-Molecule stimulators of NRF1 transcriptional activity. ChemBioChem.

[168] Kaneko M., Koike H., Saito R., Kitamura Y., Okuma Y., Nomura Y. (2010). Loss of HRD1-mediated protein degradation causes amyloid precursor protein accumulation and amyloid-β generation. J. Neurosci..

[169] Li F., Gao B., Dong H., Shi J., Fang D. (2015). Icariin induces Synoviolin expression through NFE2L1 to protect neurons from ER stress-induced apoptosis. PLoS ONE.

[170] Zhao R., Hou Y., Xue P., Woods C.G., Fu J., Feng B., Guan D., Sun G., Chan J.Y., Waalkes M.P. (2011). Long isoforms of NRF1 contribute to arsenic-induced antioxidant response in human keratinocytes. Environ. Health Perspect..

[171] Yip M.C.J., Bodnar N.O., Rapoport T.A. (2020). Ddi1 is a ubiquitin-dependent protease. Proc. Natl. Acad. Sci. USA.

[172] Lehman N.L. (2009). The ubiquitin proteasome system in neuropathology. Acta Neuropathol..

[173] Galves M., Rathi R., Prag G., Ashkenazi A. (2019). Ubiquitin signaling and degradation of aggregate-prone proteins. Trends Biochem. Sci..

